# Assessing the Efficacy of the Hinotori Robotic System in Prostate Cancer Surgery: A Systematic Review and Meta-Analysis of Observational Studies

**DOI:** 10.7759/cureus.106739

**Published:** 2026-04-09

**Authors:** Yousra Mhande, Laila Merghat, Youssef M'hamdi, Anass Guetbach

**Affiliations:** 1 Urology, Hassan II University, Casablanca, MAR

**Keywords:** efficacy, hinotori surgical robot system, robot-assisted radical prostatectomy, robotic assited surgery, s: prostate cancer

## Abstract

Prostate cancer remains a major health concern worldwide. Recently, several robotic systems have been introduced for surgical treatment in urology, demonstrating promising results and gaining clinical acceptance. Among these, a surgical robot developed in Japan has become a notable option for robot-assisted procedures. This study aims to evaluate the efficacy and safety of Hinotori robot-assisted radical prostatectomy (h-RARP) in the management of prostate cancer. A systematic review and meta-analysis of observational studies involving adult prostate cancer patients undergoing h-RARP were conducted. Literature searches were performed across PubMed, Embase, the Cochrane Library, Web of Science, and Google Scholar. Study bias was assessed using the Risk of Bias Assessment Tool for Non-Randomized Intervention Studies (ROBINS-I) tool and the Newcastle-Ottawa Scale. Meta-analyses were performed with Comprehensive Meta-Analysis software (Biostat, Inc., Englewood, NJ, USA). Twelve studies, including 1,570 patients, were analyzed, of whom 716 underwent h-RARP. Compared to the comparison group undergoing robot-assisted radical prostatectomy (C-RARP), h-RARP was associated with significantly longer console time (mean difference [MD] = 29.17 minutes; 95% confidence interval [CI]: 15.50 to 42.83) and operative time (MD = 24.98 minutes; 95% CI: 7.33 to 42.64). No significant differences were observed in postoperative and oncological outcomes. Lymph node dissection was performed in 31% of cases, and nerve-sparing was achieved in 29.3%. The positive surgical margin rate was 26.8%, while biochemical recurrence at 12 months occurred in 8.6% of h-RARP patients. In summary, while h-RARP appears to involve longer console and operative times compared to the comparison group (C-RARP), further research is required to fully clarify its clinical benefits.

## Introduction and background

Prostate cancer represents a substantial global health burden, with incidence and mortality ranking second and fifth, respectively, among cancers affecting men worldwide in 2020 [1].

Radical prostatectomy is a common therapeutic option for individuals diagnosed with localized prostate cancer [2]. While open and laparoscopic approaches were historically used, robot-assisted radical prostatectomy (RARP) is now the most popular surgical technique. This transition parallels the widespread integration of robot-assisted surgery in urologic oncology, encompassing cancers of the prostate, kidney, and bladder. To date, the da Vinci surgical system (DVSS; Intuitive Surgical, Sunnyvale, CA, USA) has dominated the field; several novel robotic platforms have recently emerged [3,4].

The Hinotori Surgical Robot System (HSRS) is a recently developed robot-assisted surgical platform from Japan, produced by Medicaroid Corporation (Kobe, Hyogo, Japan) with support from Kawasaki Heavy Industries, Ltd, and Sysmex Corporation. The system comprises a surgeon cockpit, an operation unit, and a monitor cart. Within the cockpit, the arrangement of the three-dimensional viewer, hand controllers, and foot controls closely resembles that of the DVSS, although minor variations in button and pedal placement are present. However, the HSRS features compact eight-axis operation arms and a docking-free design that allows software-defined control of the instrument fulcrum and reduces inter-arm interference. These structural innovations prompt consideration of whether differences in system architecture may affect surgical performance and quality [5].

Accordingly, this meta-analysis seeks to assess the safety and effectiveness of the HSRS in prostate cancer surgery by comparing perioperative, postoperative, and oncological outcomes with those reported for other robotic platforms.

## Review

Methods and materials

Study Design and Registration

This systematic review and meta-analysis was carried out following the guidelines of the Preferred Reporting Items for Systematic Reviews and Meta-Analyses (PRISMA) statement [6]. The study protocol was registered with PROSPERO (CRD420253032193), and the research question was developed based on the PICOS framework (Population, Intervention, Comparator, Outcome, Studies).

Eligibility Criteria

All observational studies (prospective and retrospective) involving adult male patients over 18 years old with prostate cancer who underwent prostatectomy using the HSRS, with or without comparison to other robotic systems, reporting perioperative, postoperative, and oncological outcomes, were included. We excluded randomized clinical trials, case reports, case series, non-original articles (e.g., reviews, commentaries, and posters), studies involving procedures other than Hinotori RARP (h-RARP), and those with overlapping patient data. No language or date limits were applied.

Search Strategy

**Figure 1:**
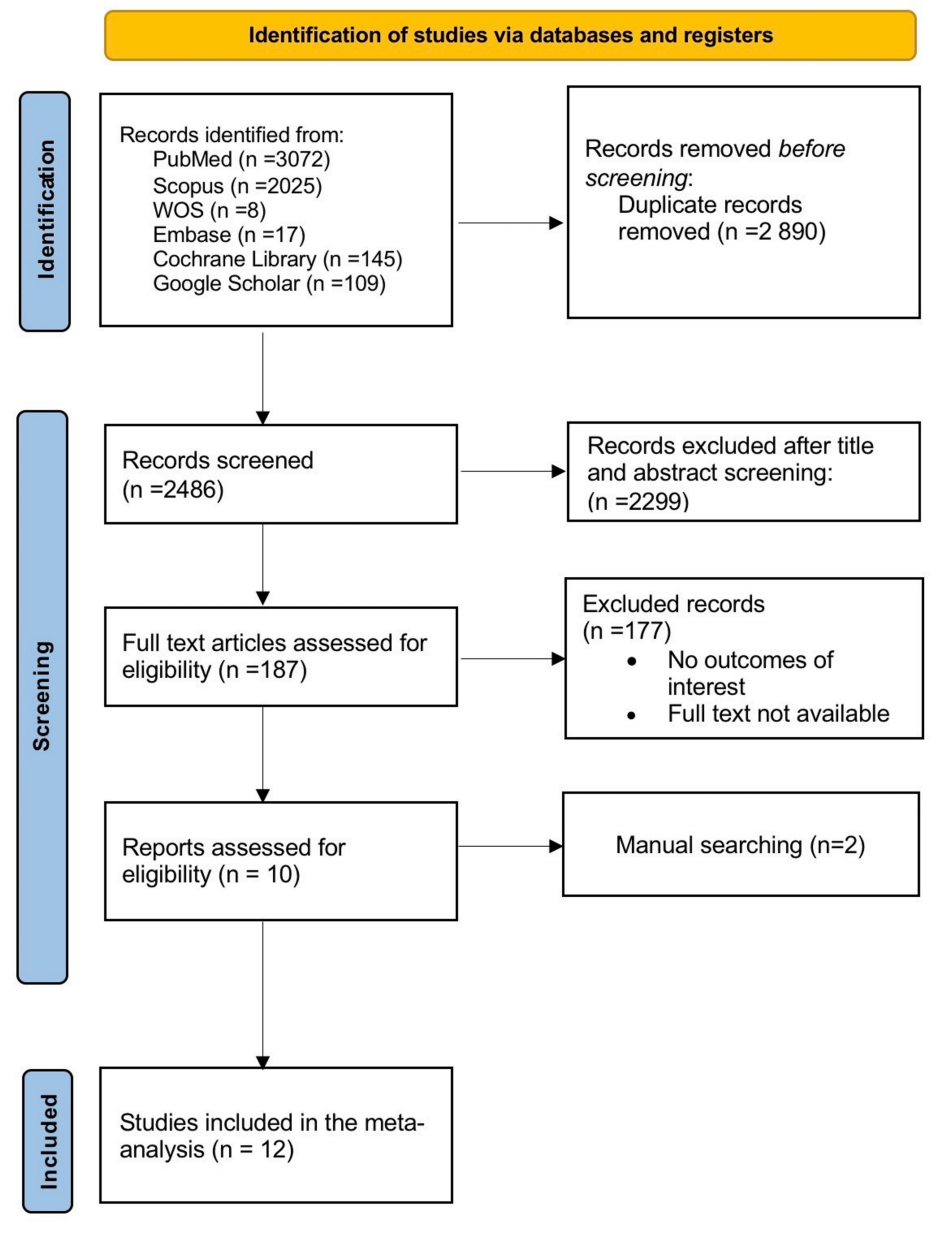
Preferred Reporting Items for Systematic Reviews and Meta-Analyses (PRISMA) diagram illustrating the results of the literature search and screening stages. WOS: Web of Science

A comprehensive search was carried out across multiple electronic databases, including PubMed, Scopus, the Cochrane Library, Embase, Web of Science, and Google Scholar. Details of each database are shown in the PRISMA flow diagram (Figure 1) [7]. Databases were searched from 15 April 2025 to 18 September 2025.

Our search strategy targeted studies contrasting prostatectomy with HSRS in the treatment of prostate cancer, using the following terms and Medical Subject Headings (MeSH): (("Robotics"[Mesh] OR "Robotic Surgical Procedures"[Mesh]) AND ("Prostatic Neoplasms"[Mesh]) AND ("Prostatectomy"[Mesh] OR "Surgical Procedures, Operative"[Mesh])), and (("Hinotori surgical robotic system" OR "Hinotori" OR "Hinotori Surgical Robot") AND ("Prostatic Neoplasms" OR "Prostatic cancer" OR "prostate cancer") AND ("Prostatectomy" OR "radical prostatectomy" OR "surgical treatment")). No language limitations were imposed. Results from each database were imported into EndNote X9, and duplicates were removed.

Study Selection

Literature searches were conducted according to the predefined criteria. Two authors independently reviewed the titles and abstracts to determine the eligible articles. Full texts of articles meeting the eligibility criteria were then reviewed by two authors independently. Studies were excluded if they lacked outcomes of interest or if the full text was unavailable. Discrepancies between authors were managed by consensus or through involvement of a third author.

Data Collection and Data Extraction

The data collection sheet was created by the corresponding author in Microsoft Excel (Redmond, WA, USA) and divided into three sections: the first recorded details about studies and participants, the second evaluated the perioperative and postoperative outcomes, and the third focused on oncological outcomes. Two authors independently conducted a thorough assessment of the relevant articles to extract necessary data.

Data extracted in section one consisted of the study title, year of publication, country of origin, sample size, and whether a comparison was made. Patient characteristics were collected for each robotic system, along with duration of follow-up and National Comprehensive Cancer Network (NCCN) risk classification. Perioperative outcomes included operative time, estimated blood loss (EBL), and console time, while postoperative outcomes focused on length of hospital stay and the occurrence of major complications within 30 and 90 days. Oncological outcomes of interest were positive surgical margins (PSM) and biochemical recurrence. In addition, data on the number of patients undergoing nerve-sparing procedures and lymph node dissection (LND) were also extracted.

Risk of Bias Assessment

The quality assessment of the selected studies was conducted independently by two authors using both the Newcastle-Ottawa Scale (NOS) [8] and the Risk of Bias Assessment Tool for Non-Randomized Intervention Studies (ROBINS-I) [9].

Data Synthesis and Analysis

Statistical analyses were performed using Comprehensive Meta-Analysis software (version 3.0; Biostat, Inc., Englewood, NJ, USA). We combined the results across studies to calculate overall mean differences (MDs) for continuous outcomes, odds ratios (ORs) for binary outcomes, and the prevalence of selected perioperative (nerve-sparing and LND) and oncological outcomes (PSM and biochemical recurrence) following h-RARP, each with 95% confidence intervals, using a random-effects model. Heterogeneity was considered significant if the p-value was less than 0.10 and was quantified using the I² statistic. Publication bias was assessed through analysis of funnel plot symmetry and Egger’s test. Sensitivity analyses were performed using a leave-one-out approach by sequentially excluding studies assessed as at serious risk of bias according to the ROBINS-I tool.

Before conducting the meta-analysis, reported data, specifically medians and interquartile ranges, were converted into means and standard deviations using validated equations [10]. For studies comparing h-RARP with two robotic systems, we followed the Cochrane Handbook guidance for handling multi-arm studies to avoid unit-of-analysis errors. Intervention groups were combined to form a single pairwise comparison, and pooled sample sizes, means, and standard deviations were calculated according to section 6.5 of the Cochrane Handbook [11,12].

Results

Literature Search and Screening Results

Based on the predefined search strategy, 5,376 articles were initially selected, of which 2,486 remained after duplicate removal. An initial screening based on titles and abstracts excluded 2,299 publications. Twelve studies [5,13-23], including 10 from full-text screening [5,13-17,20-23] and two from manual search [18,19] (Figure 1), provided data on 1,570 participants diagnosed with prostate cancer. Of these studies, eight focused on a comparison between h-RARP and the da Vinci system [5,13-19], one extended the comparison to include the Hugo system [20], while three reported no comparative analysis [21-23].

Basic Characteristics

All studies were carried out in Japan [5,13-23], and the year of publication ranged from 2022 to 2025. Among 1,570 participants with prostate cancer, 716 patients were treated with the Hinotori robotic system, while 838 patients were operated on with the da Vinci system, and one study [20] reported 16 patients treated with the Hugo system. All the reported studies were retrospective cohorts, and the characteristics table showed no difference between h-RARP and the compared system. Detailed information on the study population is provided in Table 1.

**Table 1:** Baseline characteristics table of the included studies. Abbreviations: RC: Retrospective cohort; h-RARP: The provided data of patients who received Hinotori robot-assisted radical prostatectomy; C-RARP: The provided data of patients who received radical prostatectomy with comparator robot-assisted systems; SD: standard deviation; NA: Not available; BMI: Body mass index; PSA: prostate-specific antigen.

Study	Year	Country	Cohort type	Sample Size (n)		Age Mean (SD) or Median		BMI Mean (SD) or Median		PSA Mean (SD) or Median		Prostate volume Mean (SD) or Median		Follow-up (months)		NCC Risk classification, n (%)					
																						Low		Intermediate		High/Very High	
																												h-RARP	C-RARP	h-RARP	C-RARP	h-RARP	C-RARP	h-RARP	C-RARP	h-RARP	C-RARP	h-RARP	C-RARP	h-RARP	C-RARP	h-RARP	C-RARP	h-RARP	C-RARP
																Miyamoto et al. [5]	2024	Japan	RC	42	126	67.33 (10.74)	69 (6.75)	23.9 (3.37)	23.53 (2.24)	7.36 (3.76)	8.03 (4.42)	30.03 (10.66)	30.33 (10.79)	NA		NA		NA		NA	
																																						Nakayama et al. [13]	2024	Japan	RC	97	246	68.5 (7.14)	68.66 (5.22)	24.46 (3.53)	23.86 (3.05)	7.8 (4.06)	8.03 (4.39)	32.96 (10.53)	32.23 (11.33)	8.0	29.5	3 (3.1)	26 (10.6)	47(48.5)	26 (10.6	47(48.5)	105(42.7)
				Tsujioka et al. [14]	2024	Japan	RC	118	118	69.66 (6.75)	69.66 (5.25)	24.23 (3.22)	24.1 (3.15)	8.26 (4.72)	7.76 (3.37)																																													34.06 (12.38)	33.7 (11.85)	22.6	41	4(3.5)	8 (6.8)	60(50.8)	51 (43.2)	54(45)	59(50)
Kohjimoto et al. [15]	2024	Japan	RC													43	43	69.66 (6.9)	70.33 (6.9)	24.66 (3.06)	24.33 (3.06)	8.66 (3.6)	8.3 (3.6)	NA		NA		0 (0)	0 (0)	35 (81)	34 (79)	8 (9)	9 (21)																																				
																																		Sasaki et al. [16]	2024	Japan	RC	48	46	71.66 (5.34)	72.33 (5.35)	23.6 (3.13)	23.83 (3.29)	9.53 (6.11)	9 (3.44)	NA		12.8	12.3	NA		NA		NA															
				Yamada et al. [17]	2024	Japan	RC	6	14	69	63	21.7	23.2	6	6.7																																									35.3	31.9	NA		NA		NA		NA	
Obayashi et al. [18]	2025	Japan	RC													68	76	70.33 (6.81)	69 (7.55)	24.13 (2.57)	23.7 (3.1)	7.7 (2.72)	8.1 (4.83)	30.86 (14.92)	30.86 (10.27)	6	6	4 (5.9)	1(1.3)	42 (61.8)	45(59.2)	22(32.4)	30(39.5)																																
				Kanehira et al. [19]	2025	Japan	RC	42	88	67 (6.14)	66.33 (5.27)	23.36 (3.14)	23.13 (2.5)	7.56 (3.37)	8.16 (3.84)																			34.96 (23.87)	31.06 (10.77)	12		4(9.5)	4 (4.5)	21 (50)	33 (37.5)	17 (40.5)	51 (58.0)
																																												Morizane et al. [20]	2025	Japan	RC	52	97	70.66 (5.33)	70.84 (5.15)	23.26 (3.65)	23.74 (3.51)	9.4 (5.33)	9.76 (5.15)	32.96 (16.54)	28.77 (11.70)	NA		0 (0)	2 (2.06)	19 (36.54)	30 (31)	33 (63.46)	65 (67)
																																																																		Hinata et al. [21]	2022	Japan	RC	30	NA	72	NA	NA	NA	7.6	NA	NA		NA		NA		NA		NA	
Kuromatsu et al. [22]	2024	Japan	RC	88	NA	70 (22.61)	NA	25.6 (14.84)	NA	48.64 (103.84)	NA	NA		NA		NA		NA		NA																																																																			
																						Setoguchi et al. [23]	2024	Japan	RC	82	NA	68.33 (20.37)	NA	25 (12.83)	NA	19.6 (33.73)	NA	58 (69.42)	NA	3	NA	4(4.9)	NA	42(51.2)	NA	33(40.2)	NA

Risk of Bias

**Figure 2:**
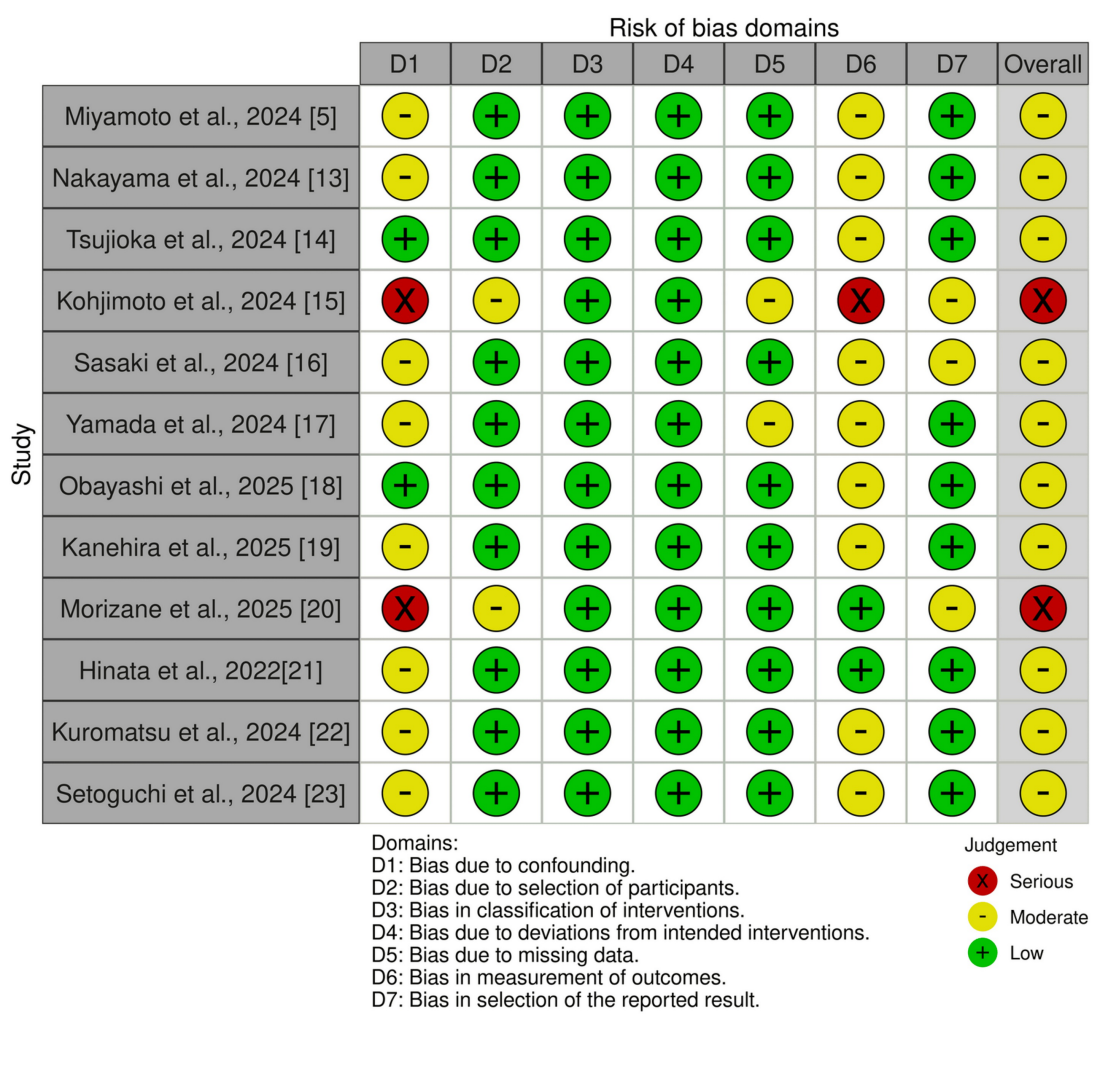
The risk of bias assessment of the included studies using Risk of Bias Assessment Tool for Non-Randomized Intervention Studies (ROBINS-I) tool.

The risk of bias of the 12 studies was assessed using both NOS and ROBINS-I [5,13-23], revealing different levels of quality (details are summarized in Table 2 and Figure 2). According to NOS, eight studies were determined to have good quality [5,13-16,18-20], while four were rated as poor quality due to issues with cohort comparability in the design or analysis [17,21-23]. The ROBINS-I evaluation yielded a moderate risk for 10 studies [5,13,14,16-19,21-23], and only two studies had a serious risk of bias because of confounding or outcome measurement bias [15,20]. None of the studies were excluded due to weakness of design or data quality.

**Table 2:** Assessment of the quality of the non-randomized studies based on the Newcastle-Ottawa Scale.

Study	Year	Selection				TOTAL - Selection	Comparability	Total –Comparability	Outcome			Total-Outcome	Total Stars (max 9)	Notes
		1	2	3	4		1		1	2	3			
Miyamoto et al. [5]	2024	1	1	1	1	4	1	1	1	1	1	3	8	Good Quality
Nakayama et al. [13]	2024	1	1	1	0	3	1	1	1	0	1	2	6	Good Quality
Tsujioka et al. [14]	2024	1	1	1	1	4	2	2	1	0	1	2	8	Good Quality
Kohjimoto et al. [15]	2024	1	1	1	0	3	2	2	1	1	0	2	8	Good Quality
Sasaki et al. [16]	2024	1	1	1	1	4	1	1	1	1	1	3	8	Good Quality
Yamada et al. [17]	2024	1	1	1	1	4	0	0	1	0	1	2	5	Poor Quality
Obayashi et al. [18]	2025	1	1	1	1	4	1	1	1	0	1	2	7	Good quality
Kanehira et al. [19]	2025	1	1	1	1	4	1	1	1	0	1	2	7	Good quality
Morizane et al. [20]	2025	1	1	1	1	4	1	1	1	1	1	3	8	Good Quality
Hinata et al. [21]	2022	1	0	1	1	3	0	0	1	0	1	2	5	Poor Quality
Kuromatsu et al. [22]	2024	1	0	1	1	3	0	0	1	0	1	2	5	Poor Quality
Setoguchi et al. [23]	2024	1	0	1	1	3	0	0	1	1	1	3	6	Poor Quality

Meta-analysis

Perioperative Outcomes

**Figure 3:**
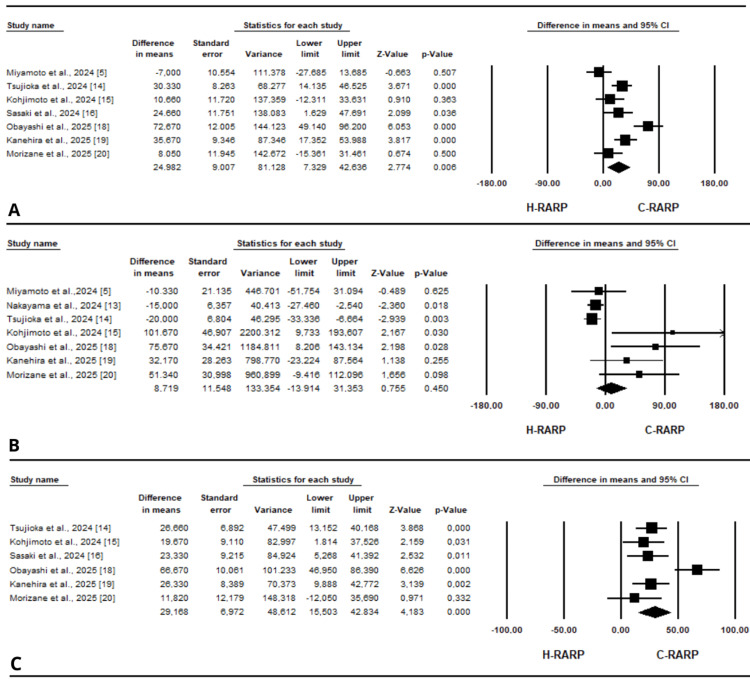
Forest plots for the included studies for studies reporting perioperative outcomes: (A) surgical or operative time, (B) estimated blood loss, (C) console time.

Surgical or operative time: Seven articles [5,14-16,18-20], rated as good to moderate quality according to the NOS (Table 2), reported operative or surgical time as an outcome. The analysis showed a significantly longer operative time for h-RARP compared to other robotic systems (MD = 24.98 minutes, 95% CI [7.33 to 42.64], p < 0.01) (Figure 3A). There was considerable heterogeneity among the included studies (I² = 80.124%, p < 0.001).

EBL: The mean difference was calculated using data from seven studies that reported EBL as medians and interquartile ranges [5,13-15,18-20], which were converted to means and standard deviations. All the studies were assessed as good quality using the NOS (Table 2). No significant differences between h-RARP and the other robotic systems were observed in EBL (MD = 8.72 mL, 95% CI [-13.91 to 31.35], p = 0.45) (Figure 3B). This finding was associated with notable heterogeneity (I² = 70.857%, p < 0.01).

Console time: Six studies reported console time and were all assessed as good quality based on the NOS [14-16,18-20] (Table 2). The pooled analysis showed a significantly longer console time, with a mean difference of 29.17 minutes (95% CI [15.50 to 42.83], p < 0.001) (Figure 3C). However, substantial heterogeneity was observed (I² = 71.671%, p = 0.003).

**Figure 4:**
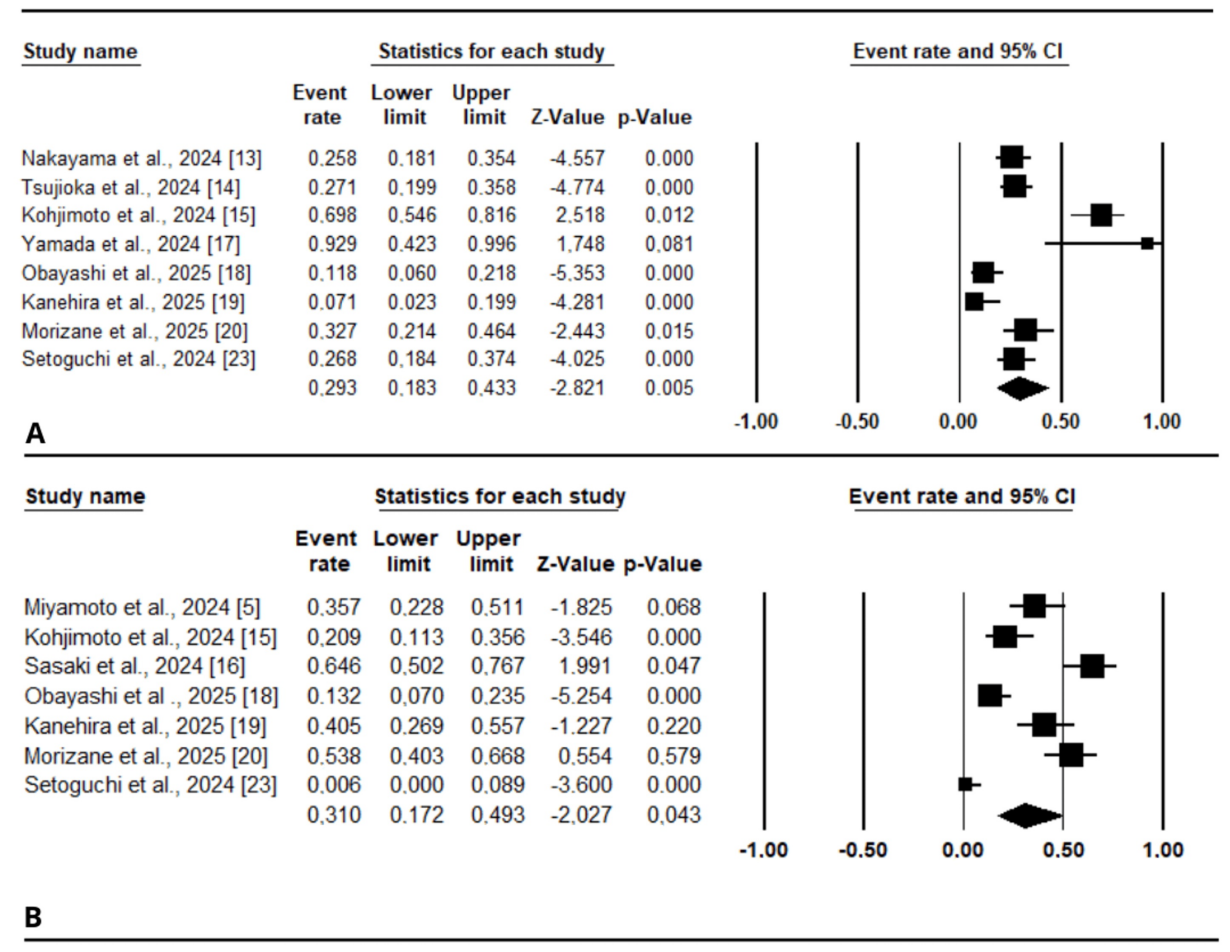
Forest plots for the included studies for studies reporting perioperative outcomes: (A) nerve sparing, (B) lymph node dissection.

Nerve sparing: Nerve sparing during h-RARP was reported in eight studies [13-15,17-20,23] and was performed in 29.3% (95% CI [0.183 to 0.433]) (Figure 4A). Significant heterogeneity was observed among the included studies (I² = 86.163%, p < 0.001). Six of the included studies were rated as good quality based on the NOS [13-15,18-20] (Table 2).

LND: LND during h-RARP was reported in seven articles [5,15,16,18-20,23]; six were assessed as good quality according to the NOS [5,15,16,18-20] (Table 2). Overall, it was performed in 31% of cases (95% CI [0.172 to 0.493]) (Figure 4B), with considerable heterogeneity among studies (I² = 87.828%, p < 0.001).

Conversion to open surgery: Five studies reported conversion to open surgery after h-RARP [5,15,18,19,23]. No conversions were required in any of the cases.

Postoperative Outcomes

**Figure 5:**
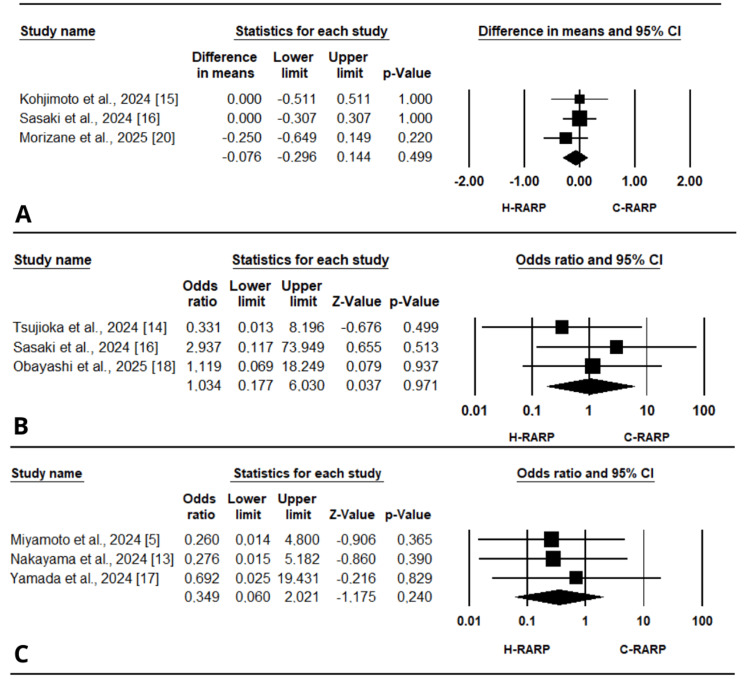
Forest plots for the included studies for studies reporting postoperative outcomes: (A) post-operative hospital stay, (B) major complications (Clavien-Dindo grade ≥3) at 30 days, (C) major complications (Clavien-Dindo grade ≥3) at 90 days.

Postoperative hospital stay: Three studies provided data on hospital stay after surgery [15,16,20]; all were assessed as good quality using the NOS (Table 2). The analysis revealed no significant difference between h-RARP and other robotic systems (MD = -0.08 days, 95% CI [-0.30 to 0.14], p = 0.50) (Figure 5A). No significant heterogeneity was observed (I² = 0%, p = 0.591).

Major complications: Major complications (Clavien-Dindo grade ≥3) at 30 days were reported in three different studies [14,16,18], while at 90 days they were reported in three other studies [5,13,17], most of which were assessed as good quality according to the NOS [5,13,14,16,18] (Table 2). The analysis showed no significant results at 30 days (OR = 1.03, 95% CI [0.18 to 6.03], p = 0.97) or at 90 days (OR = 0.35, 95% CI [0.06 to 2.02], p = 0.24) (Figure 5B, 5C). No notable heterogeneity was observed for either time point, with I² = 0% (p = 0.641) at 30 days and I² = 0% (p = 0.893) at 90 days.

Oncological Outcomes

**Figure 6:**
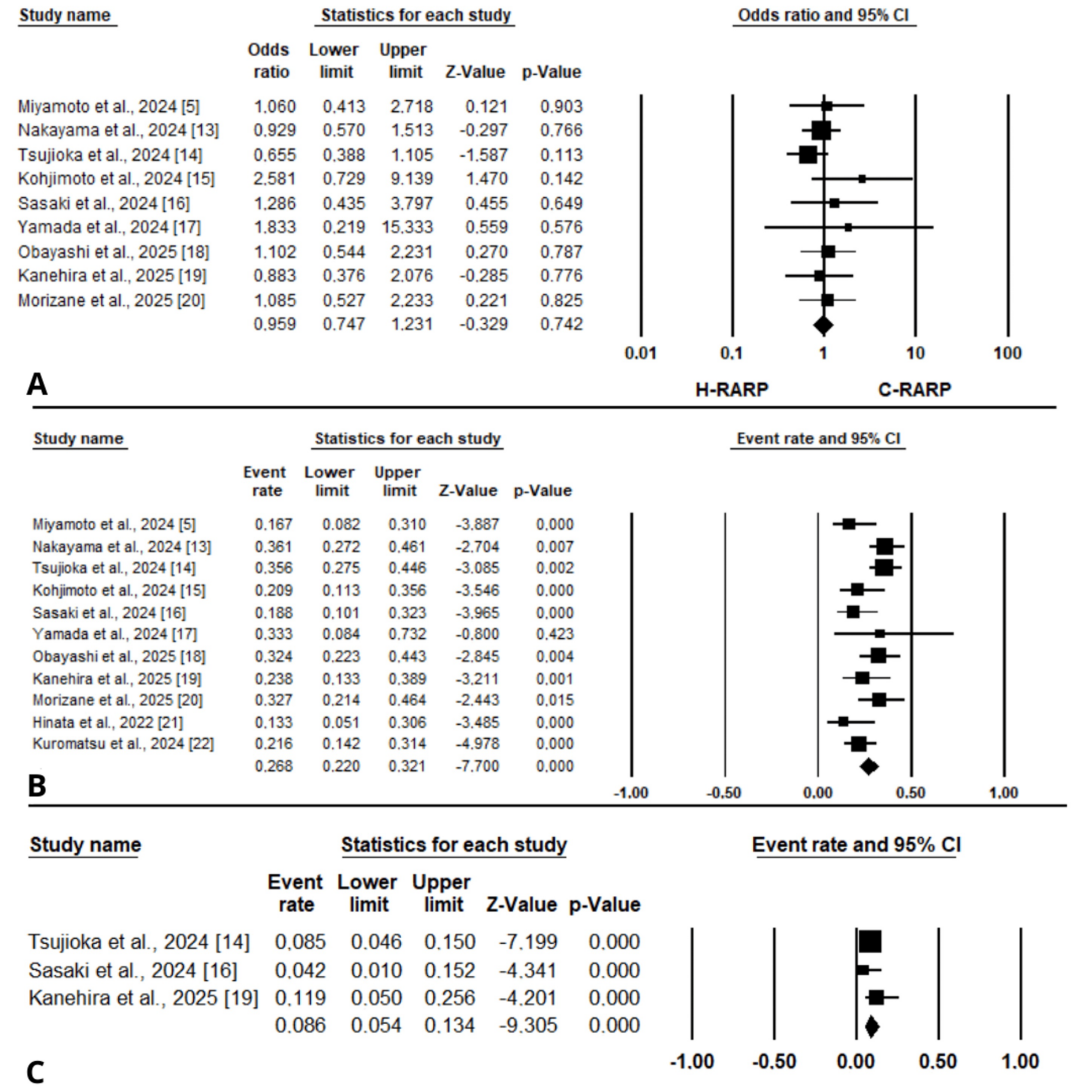
Forest plots for the included studies for studies reporting oncological outcomes: (A, B) positive surgical margins in patients with pT≥2 stage disease and (C) biochemical recurrence at 12 months.

PSM: Eleven studies reported data on PSM in patients with pT≥2 stage disease after h-RARP [5,13-22], eight of which were assessed as good quality using the NOS [5,13-16,18-20] (Table 2). The pooled PSM rate was 26.8% (95% CI [22% to 32.1%]) (Figure 6B), with significant heterogeneity among the included studies (I² = 45.617%, p = 0.049). Nine studies compared h-RARP with other robotic systems [5,13-20], and no significant difference was observed between groups (OR = 0.96, 95% CI [0.75 to 1.23], p = 0.74) (Figure 6A), with negligible heterogeneity (I² = 0%, p = 0.715).

Biochemical recurrence: Three articles [14,16,19], rated as good quality according to the NOS (Table 2), reported data on biochemical recurrence at 12 months. Following h-RARP, recurrence was observed in 8.6% of patients (95% CI [0.054 to 0.134]) (Figure 6C). No significant heterogeneity was detected among studies (I² = 0%, p = 0.423). 

The distributions of true effect sizes for all outcomes are presented in Appendices 1 and 2.

Sensitivity Analysis

To assess the robustness of our findings, we performed a leave-one-out sensitivity analysis by sequentially removing one study at a time. Across all surgical outcomes, including operative time, EBL, console time, postoperative hospital stay, and PSM, the pooled results remained consistent. No single study significantly influenced the overall effect estimates, and no meaningful changes were observed after exclusion of individual studies. These findings support the stability of the primary meta-analysis results.

Publication Bias

**Figure 7:**
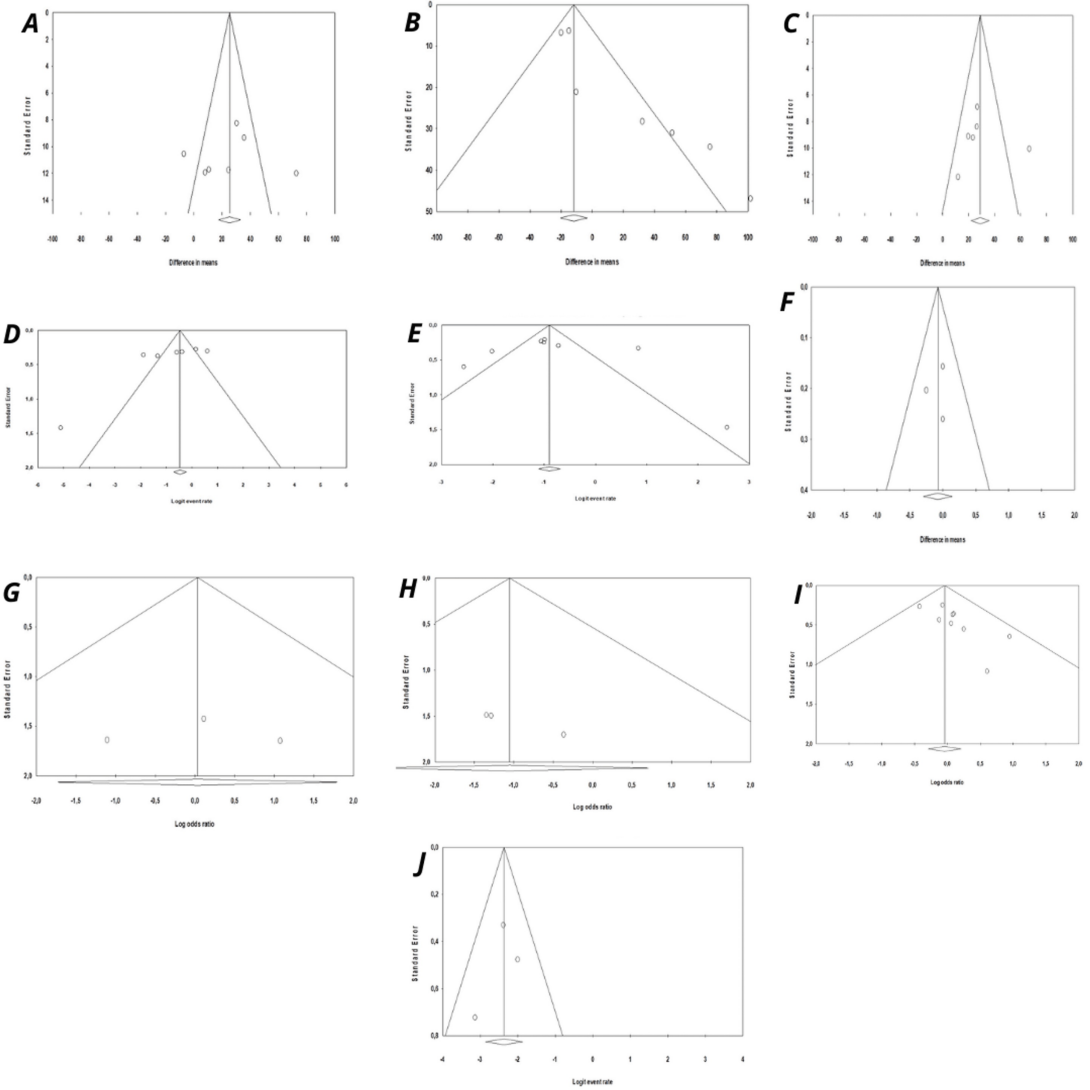
Funnel plots for the included studies for studies reporting (A) surgical or operative time, (B) estimated blood loss, (C) console time, (D) lymph node dissection, (E) nerve sparing, (F) post-operative hospital stay, (G) major complications (Clavien-Dindo grade ≥3) at 30 days, (H) major complications (Clavien-Dindo grade ≥3) at 90 days, (I) positive surgical margins in patients with pT≥2 stage disease and (J) biochemical recurrence at 12 months.

Funnel plots, presented in Figure 7, suggested possible asymmetry for EBL, PSM, and major complications at 90 days, while no clear pattern was observed for operative time, console time, LND, nerve sparing, postoperative hospital stay, major complications at 30 days, and biochemical recurrence at 12 months. However, given that all analyses included fewer than 10 studies, these findings should be interpreted with caution, as funnel plots are not considered reliable for assessing publication bias in such cases. Results of Egger’s test are presented in Appendix 3.

Discussion

This meta-analysis aimed to assess the efficacy of RARP in the context of prostate cancer, alongside other robotic systems such as Hugo and da Vinci. Consistent with prior reviews [24,25], our analysis revealed significant results related to operative time and console time, with longer durations observed for both. However, no significant differences were observed in EBL, postoperative hospital stay, major complications within 30 or 90 days, and PSM between the h-RARP and the other robotic systems. In addition, analysis of h-RARP showed an LND rate of 31% and nerve sparing in 29.3% of cases, with PSM in 26.8% and biochemical recurrence in 8.6%.

These findings suggest that both h-RARP and other established robotic systems in urology are effective in the treatment of prostate cancers.

Robotic systems, especially in the field of urology, enhance surgical performance and facilitate surgery compared with traditional methods by providing high-resolution 3D imaging and greater operational freedom, which improve surgical outcomes and enable functional preservation, leading to a better quality of life [26]. The h-RARP is a newly developed robotic-assisted surgical system from Japan, and is regarded as a competitor to the da Vinci and Hugo systems [15,16]. It consists of three main units: an operation unit, a surgical cockpit, and a vision unit. The system offers eight freedom axes and an anti-shake mechanism, along with a computerized control used to prevent collisions between the robotic arms. The safety of this system was first evaluated in a preclinical study using fresh cadavers and live porcine models between 2019 and 2020, followed by an initial human trial conducted between 2020 and 2021 [21].

The experience of this new system is not limited to radical prostatectomy but extends to partial or radical nephrectomy, nephroureterectomy, adrenalectomy, cystectomy, and cystectomy with intracorporeal urinary diversion, thereby enhancing its relevance in urological surgery [5].

The evolution of robotic surgery systems began in the 2000s in the United States with the introduction of the da Vinci Standard. This endoscopic surgical system consisted of three robotic arms, a laparoscope, and an operating console equipped with a pedal unit for controlling different energy sources. Since then, the system has evolved continuously, leading to the development of different models: da Vinci S, da Vinci Si, da Vinci Xi, and, most recently, the da Vinci SP introduced in 2022 as a single-port surgical platform [26]. In August 2020, HSRS received regulatory approval in Japan. It differs from the da Vinci system in several ways, notably by providing eight axes of movement for each robotic arm, which helps prevent collisions between different arms during surgery. In addition, dedicated software allows the pivot point to be set without attaching the trocar, creating more space around the ports and reducing excessive tension on the tissue. Moreover, the 3D visual interface in the surgeon’s cockpit can be adjusted for comfort, promoting a relaxed posture [27]. As a result of its performance and ease of handling, Hinotori has become increasingly popular in Japan, particularly in urology, along with gynecology and gastroenterology [26].

The Hugo robotic-assisted system is considered one of the most recently developed surgical robots. It consists of four independent arm carts, each with six joints to extend its range of motion, and uses dedicated 3D glasses for head-tracking [28].

Success in surgery depends not only on the technology used but also on the surgeon’s level of training, which is reflected in the learning curve. The learning curve in robotic surgery is finite and achievable, with most surgeons showing noticeable improvements in efficiency within 10 to 30 cases. Fundamental console skills are often acquired early, within the first 10 procedures, while optimization of operating room workflow, including docking and team coordination, develops more gradually. Studies show a progressive reduction in operative time after 20 cases, with performance stabilizing around 30 cases, reaching levels comparable to those of experienced laparoscopic surgeons. Additionally, the learning phase is not associated with higher complication rates when appropriate supervision and careful case selection are maintained, supporting the safety and feasibility of structured robotic training [29].

The integration of robotic systems into surgical fields has profoundly transformed medical practice. However, these systems remain expensive in terms of acquisition and maintenance [27].

This study has several strengths, including a comprehensive literature search using large databases and the application of rigorous selection criteria, which enhance the reliability of the findings. No language restrictions were applied, allowing for more complete reporting of the available evidence.

However, some limitations should be noted. The small sample size of the included studies may increase the risk of bias. Variability in the risk of bias across studies could also affect the strength of the conclusions. In addition, limited reporting of certain outcomes, including postoperative hospital stay, major complications, and biochemical recurrence, restricted the analyses. Finally, as all included studies were conducted in a single country, the generalizability of the findings to other populations may be limited.

Future studies should include more diverse populations, report a broader range of outcomes, and use longer follow-up periods to better assess long-term postoperative outcomes and recurrence.

## Conclusions

This systematic review and meta-analysis suggest that the Hinotori robotic system is a safe and effective option for radical prostatectomy, possibly offering a good alternative to current robotic systems. By combining data from 12 studies, which included 1,570 patients, this research provides the most complete and up-to-date summary of the available evidence. It also reports the first pooled oncological outcomes for Hinotori-assisted surgery, including PSM rates and 12-month biochemical recurrence, while considering additional surgical indicators such as nerve-sparing, LND, and conversion rates. However, further well-designed studies are still needed to better confirm its clinical value.

## Figures and Tables

**Figure 8:**
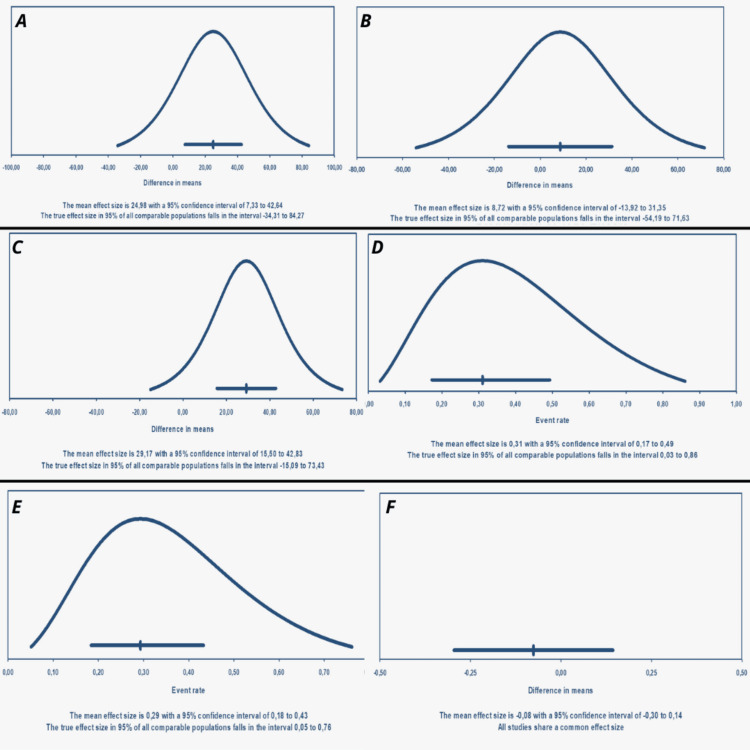
Distribution of true effect size for the included studies for studies reporting (A) surgical or operative time, (B) estimated blood loss, (C) console time, (D) lymph node dissection, (E) nerve sparing, (F) post-operative hospital stay.

**Figure 9:**
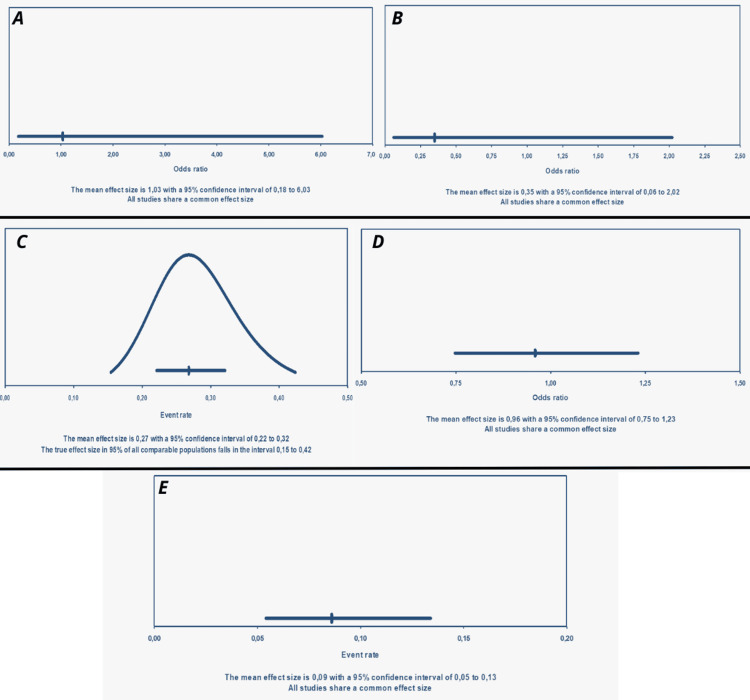
Distribution of true effect size for the included studies for studies reporting (A) major complications (Clavien-Dindo grade ≥3) at 30 days, (B) major complications (Clavien-Dindo grade ≥3) at 90 days, (C,D) positive surgical margins in patients with pT≥2 stage disease and (E) biochemical recurrence at 12 months.

## Appendices

Appendix 1 

Appendix 2  

Appendix 3  

**Table 3:** Egger's Test of the Intercept for Publication Bias. Note: The 1-tailed p-value is recommended for interpretation. EBL: estimated blood loss; LND: lymph node dissection; PSM: positive surgical margins

Outcome	Intercept (B0)	95% CI	t-value	df	1-tailed p-value	2-tailed p-value
Operative time	-0.94970	[-17.77641, 15.87701]	0.14508	5	0.44516	0.89031
EBL	2.51527	[1.31113, 3.71940]	5.36958	5	0.00151	0.00302
Console time	0.71344	[-13.19857, 14.62545]	0.14238	4	0.44683	0.89366
LND	-5.86263	[-13.20902, 1.48376]	2.05140	5	0.04774	0.09547
Nerve sparing	0.71440	[-5.69946, 7.12826]	0.27255	6	0.39717	0.79434
Post-operative hospital stay	-0.65888	[-36.90423, 35.58647]	0.23098	1	0.42774	0.85549
Major complications (Clavien–Dindo ≥3) at 30 days	-0.38481	[-101.31961, 100.54998]	0.04844	1	0.48459	0.96918
Major complications (Clavien–Dindo ≥3) at 90 days	4.53072	[3.01030, 6.05113]	37.86348	1	0.00840	0.01681
PSM	1.56909	[0.26730, 2.87087]	2.85016	7	0.01234	0.02468
Biochemical recurrence at 12 months	-1.31112	[-29.26911, 26.64688]	0.59587	1	0.32895	0.65790
